# Overexpression of *Dlx2* enhances osteogenic differentiation of BMSCs and MC3T3-E1 cells via direct upregulation of *Osteocalcin* and *Alp*

**DOI:** 10.1038/s41368-019-0046-1

**Published:** 2019-03-18

**Authors:** Jianfei Zhang, Wenbin Zhang, Jiewen Dai, Xudong Wang, Steve Guofang Shen

**Affiliations:** Department of Oral & Cranio-maxillofacial Surgery, Shanghai Ninth People’s Hospital, Shanghai Jiao Tong University School of Medicine, Shanghai Key Laboratory of Stomatology, No. 639 Zhizaoju Road, Shanghai, China

**Keywords:** Mechanisms of disease, Bone development, Mechanisms of disease

## Abstract

Genetic studies have revealed a critical role of Distal-homeobox (Dlx) genes in bone formation, and our previous study showed that *Dlx2* overexpressing in neural crest cells leads to profound abnormalities of the craniofacial tissues. The aim of this study was to investigate the role and the underlying molecular mechanisms of *Dlx2* in osteogenic differentiation of mouse bone marrow stromal cells (BMSCs) and pre-osteoblast MC3T3-E1 cells. Initially, we observed upregulation of Dlx2 during the early osteogenesis in BMSCs and MC3T3-E1 cells. Moreover, *Dlx2* overexpression enhanced alkaline phosphatase (ALP) activity and extracellular matrix mineralization in BMSCs and MC3T3-E1 cell line. In addition, micro-CT of implanted tissues in nude mice confirmed that *Dlx2* overexpression in BMSCs promoted bone formation in vivo. Unexpectedly, *Dlx2* overexpression had little impact on the expression level of the pivotal osteogenic transcription factors *Runx2*, *Dlx5*, *Msx2*, and *Osterix*, but led to upregulation of *Alp* and *Osteocalcin* (*OCN*), both of which play critical roles in promoting osteoblast maturation. Importantly, luciferase analysis showed that *Dlx2* overexpression stimulated both *OCN* and *Alp* promoter activity. Through chromatin-immunoprecipitation assay and site-directed mutagenesis analysis, we provide molecular evidence that Dlx2 transactivates *OCN* and *Alp* expression by directly binding to the Dlx2-response *cis*-acting elements in the promoter of the two genes. Based on these findings, we demonstrate that *Dlx2* overexpression enhances osteogenic differentiation in vitro and accelerates bone formation in vivo via direct upregulation of the *OCN* and *Alp* gene, suggesting that Dlx2 plays a crucial role in osteogenic differentiation and bone formation.

## Osteogenesis: Dlx2 gene plays key role in bone development

The distal-less homeobox (*Dlx*) gene family is related to various features of bone development, and the *Dlx2* member of that family has been found to play a crucial role in bone formation. A team headed by Steve Guofang Shen at the Shanghai Jiao Tong University School of Medicine in China investigated the function of *Dlx2* in osteogenic (bone development) differentiation of mouse bone marrow stromal cells (BMSCs) and MC3T3-E1 cells (precursors of osteoblasts, the major cellular component of bone). The team found that overexpression of *Dlx2* promotes osteogenic differentiation in vitro and accelerates bone formation in vivo by enhancing *Osteocalcin* and *Alp* genes (both of which play critical roles in promoting osteoblast maturation). The authors conclude that their results suggest a promising future strategy for treating bone defects where BMSCs overexpress *Dlx2*.

## Introduction

The distal-less homeobox (Dlx) gene family consists of six members (*Dlx1*, *Dlx2*, *Dlx3*, *Dlx5*, *Dlx6*, and *Dlx7*); these members are expressed in a complex pattern in the first and second branchial arch region.^1^ Dlx1/2 regulates the development of the upper jaw, while Dlx5/6 regulates lower jaw development.^2^ Together with other homeobox proteins, the Dlx family regulates osteoblast differentiation. As one of the key transcription factors regulating osteogenic differentiation, Dlx5 stimulates two other key transcription factors, Runx2 and Osterix (Osx), which sequentially induce expression of bone markers such as *Osteocalcin* (*OCN*) and *Alkaline* *Phosphatase* (*ALP*). The expression of *Dlx5* is induced by bone morphogenetic protein-2 (BMP-2).^3^ Msx2, another homeobox gene and a key regulator of osteogenic differentiation, represses the expression of *Alp* by directly binding to its promoter, while Dlx5 activates its expression by interfering with the ability of Msx2.^4^ Thus, Dlx5 coordinates with Msx2 to regulate osteogenic differentiation due to their reciprocal ability to compete with each other.

Sharing strong sequence similarity with Dlx5, Dlx2 has been shown to play a crucial role in craniofacial skeletal development.^5^ *Dlx2* is upregulated in the central area of the first branchial arch during days 9.5 and 10.5 of embryonic development in mice. This upregulation of *Dlx2* is important for the differentiation and development of the primordium, as it leads to the development of the maxillofacial skeletal pattern.^6^ Given that Dlx5 controls osteogenic differentiation,^7^ it is reasonable to speculate that Dlx2 might be involved in this process. So far, only a few studies have reported that *Dlx2* overexpression increases the osteogenic differentiation potential of pre-osteoblast cells.^8^ However, how Dlx2 regulates osteogenic differentiation and the underlying cellular and molecular mechanisms remain unknown.

In a previous study, we found that elevated *Dlx2* expression led to midfacial development defects, nasal deformities, premaxillary bony deficiency, and spine deformities.^9^ Thus, it is crucial to examine how *Dlx2* overexpression leads to abnormal bone formation both in vitro and in vivo. To investigate the role of Dlx2 during osteogenic differentiation both in vitro and in vivo, we used mouse bone marrow stromal cells (BMSCs) in our study, as the ability of BMSCs to differentiate toward adipogenic, chondrogenic, and osteogenic cell lineages has been characterized extensively in vivo and in vitro by various researchers.^10^ Osteogenic differentiation of BMSCs can be assayed in vitro by ALP and Alizarin red staining and in vivo by transplantation assays.^11,12^ Therefore, mouse BMSCs are suitable for investigating the effect of *Dlx2* overexpression on osteogenesis both in vitro and in vivo. Murine osteoblastic cell line MC3T3-E1 cells were also chosen to verify the effect of *Dlx2* overexpression on osteogenesis in vitro.

Initially, we observed the upregulation of *Dlx2* in both mouse BMSCs and MC3T3-E1 cells during osteogenic differentiation. Moreover, forced overexpression of *Dlx2* led to enhanced osteogenic differentiation potential of both BMSCs and MC3T3-E1 cells in vitro, and accelerated bone formation in vivo. These findings prompted us to explore the underlying mechanisms. To our surprise, we found that *Dlx2* overexpression had no significant effect on the expression levels of *Dlx5*, *Msx2*, *Runx2*, and *Osx*, but led to upregulation of *Alp* and *OCN* in BMSCs and MC3T3-E1 cells. Considering the fact that Alp promotes the early stage of osteogenic differentiation and OCN accelerates the late stage, we next analyzed the promoter of *OCN* and *Alp* through luciferase-reporter assay and chromatin-immunoprecipitation (ChIP) analysis, and found that *Dlx2* transcriptionally regulated *OCN* and *Alp* expression by directly binding to their promoters. Taken together, our data demonstrates for the first time that *Dlx2* overexpression enhances the early stage of osteogenic differentiation via direct upregulation of *Alp*, and promotes the late stage of osteogenic differentiation via direct upregulation of OCN.

## Results

### Endogenous Dlx2 expression in BMSCs and MC3T3-E1 cells during osteogenesis

**Fig. 1:**
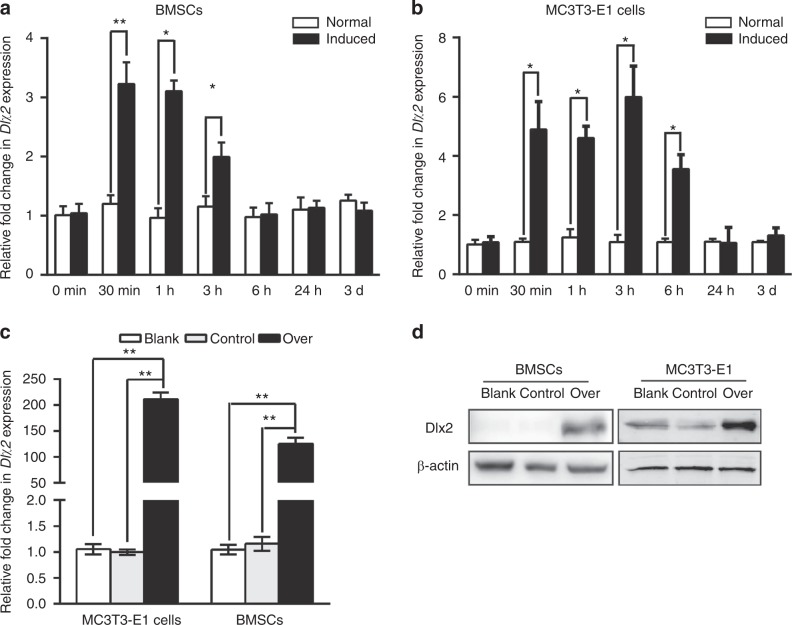
Analysis of *Dlx2* expression by RT-qPCR in BMSCs and MC3T3-E1 cells upon osteogenic induction. Endogenous expression of Dlx2 in BMSCs (**a**) and MC3T3-E1 cells (**b**) at different time points upon osteogenic induction. Relative transcript levels of *Dlx2* at each time point were quantified by RT-qPCR and normalized with a house-keeping gene *Gapdh*. Student’s *t* tests were used to determine statistical significance; *n* = 3. Error bars represent SDs. *P < 0.05; **P < 0.01. **c** *Dlx2* expression in BMSCs and MC3T3-E1 cells was evaluated with RT-qPCR. Gene expression was normalized with *Gapdh*, and statistical significance was determined as described in **a**. **d** Protein levels of Dlx2 in BMSCs and MC3T3-E1 cells were measured by Western blot analysis. Blank, BMSCs/MC3T3-E1 cells; control, Lenti-CTRL transduced BMSCs/MC3T3-E1 cells; over, Lenti-DLX2 OE transduced BMSCs/MC3T3-E1 cells. β-Actin was used as an internal control

First, we examined the levels of *Dlx2* expression upon osteogenic induction in mouse BMSCs and MC3T3-E1 cells. Quantitative reverse transcription polymerase chain reaction (RT-qPCR) results showed that when BMSCs were exposed to osteogenic-inducing medium (OIM), *Dlx2* expression was upregulated within 0.5 and 3 h after induction (Fig. 1a). However, after 7- or 14-day culture in OIM, these cells express similar mRNA level of *Dlx2* with the cells cultured in normal culture medium (data not shown). In addition, western blot analysis with an anti-Dlx2 antibody detected only a very weak signal of Dlx2 protein in BMSCs cultured both in normal medium and OIM for 3 h. This could be explained by the low protein level of endogenous Dlx2 in BMSCs. Supporting this notion is the finding that the endogenous protein level of other Dlx proteins, such as Dlx5, is also quite low in BMSCs.^13^

The expression pattern of *Dlx2* in MC3T3-E1 cells was similar with that in BMSCs. The mRNA level of *Dlx2* in MC3T3-E1 cells was upregulated within and the first 6 h after osteogenic induction (Fig. 1b), but showed no differences with that in the cells cultured in normal culture medium after culture for 7 or 14 days (data not shown). Consistently, previous study in stem cells from apical papilla (SCAP) also showed a similar expression pattern that Dlx2 was upregulated within the first few hours upon osteogenic induction, and its expression then showed no significant difference with that in cells grown in normal cell culture medium. This reduction of *Dlx2* expression in the induced group could be explained by the regulation of mRNA stability by microRNAs (miRNAs). Latest study reveals that Dlx2 is a target gene of the miRNA, miR-185-5p, and its expression is negatively regulated by Dlx2.^14^ The initial upregulation of Dlx2 may stimulate the expression of downstream target genes, which induces miRNA expression and sequentially exert a feedback on Dlx2 expression. In addition, western blot analysis detects only weak expression of Dlx2 in MC3T3-E1 cells treated either with OIM or normal culture medium, consistent with the endogenous protein level of Dlx2 in BMSCs. Together, these results demonstrate that *Dlx2* was upregulated during early osteogenesis in mouse BMSCs and MC3T3-E1 cells.

### Forced overexpression of *Dlx2* in BMSCs and MC3T3-E1 cells

Next, to investigate the effect of *Dlx2* on osteogenesis, we established *Dlx2*-overexpressing BMSCs and MC3T3-E1 cells. Cultured BMSCs or MC3T3-E1 cells were transduced with Lenti-DLX2 OE lentivirus to allow stable *Dlx2* overexpression. As a control, parallel BMSCs or MC3T3-E1 cells were transduced with Lenti-CTRL lentivirus as mock control. Dlx2 expression was then evaluated by RT-qPCR and western blot analysis. Lenti-DLX2 OE-transduced BMSCs (over) displayed much more Dlx2 transcripts (Fig. 1c) and correspondingly increased protein production (Fig. 1d) than wildtype or Lenti-CTRL-transduced BMSCs (control) did. Similarly, Lenti-DLX2 OE transduction in MC3T3-E1 cells also led to increased mRNA and protein levels of Dlx2 (Fig. 1c, d). These observations indicate that *Dlx2* was successfully overexpressed in both cell lines.

### Dlx2 overexpression enhances the osteogenic differentiation potential of BMSCs and MC3T3-E1 cells

**Fig. 2:**
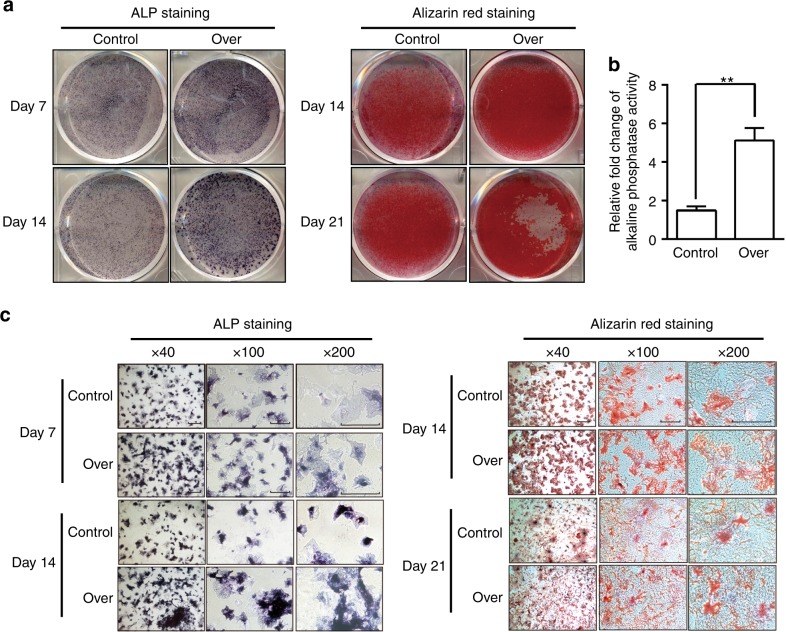
Forced overexpression of *Dlx2* enhanced osteogenesis of BMSCs in vitro. **a** ALP staining was performed on days 7 or 14 after osteogenic induction. Alizarin red staining was carried out after cells were cultured in OIM for 14 or 21 day. **b** Semi-quantitative analysis of ALP activity in *Dlx2*-overexpressing BMSCs (over) and control BMSCs (control) after 14-day culture in OIM. **c** Magnified views of ALP staining and Alizarin red staining in **a**. Scale bar = 50 μm in all the panels. Left panel, 40-fold magnified image; middle panel, 100-fold magnified image; right panel, 200-fold magnified image

To investigate whether the overexpression of *Dlx2* effects on osteogenic differentiation in vitro, we carried out ALP staining and Alizarin staining assays. Lentivirus-transduced BMSCs or MC3T3-E1 cells were cultured in OIM for 7, 14, or 21 days to assess their osteogenic differentiation potential.^14^ Interestingly, ALP staining was significantly enhanced at day 7 and 14 in the *Dlx2*-overexpressing BMSCs compared with that in control BMSCs (Fig. 2a, c). Similarly, higher ALP activity levels were observed in *Dlx2*-overexpressing BMSCs at day 14 after osteogenic induction (Fig. 2b). Furthermore, Alizarin red staining revealed that mineralization was markedly enhanced in *Dlx2*-overexpressing BMSCs during the entire culture period, especially at day 21 (Fig. 2a, c). Consistently, we also observed enhanced ALP and Alizarin red staining in *Dlx2*-overexpressing MC3T3-E1 cells (Fig. S1). Given that ALP activity is involved in the early stage of osteogenic differentiation while mineralization is involved in the late stage, we therefore proposed that Dlx2 overexpression accelerates the early stage of osteogenesis by increasing ALP activity and the late stage of osteogenesis by enhancing mineralization.

### Forced overexpression of Dlx2 in BMSCs accelerated bone formation in vivo

**Fig. 3:**
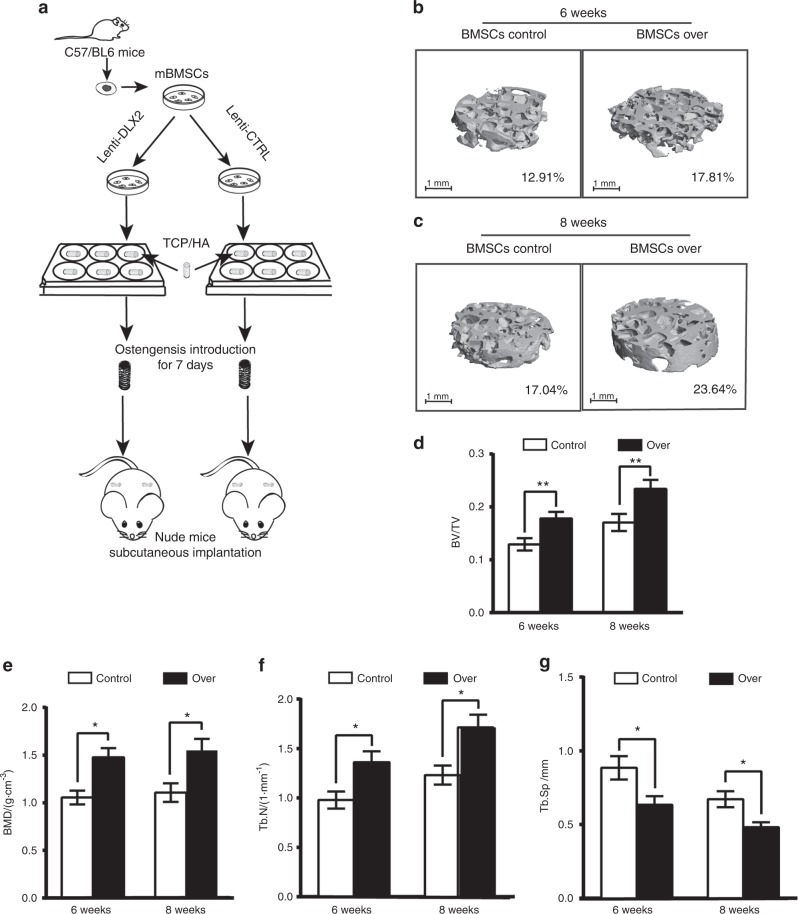
*Dlx2*-overexpressing BMSCs accelerated bone formation in vivo. **a** Schematic diagram of implantation experiments. The whole BMSCs/β-TCP constructs were obtained for micro-CT analysis 6 or 8 weeks after implantation. **b**, **c** Representative 3D reconstructed micro-CT results of the BMSCs/β-TCP constructs at weeks 6 and 8 after subcutaneous implantation. Implants were then harvested after 6 (**b**) or 8 (**c**) weeks, and were scanned by micro-CT. Scale bar = 1 μm. Average BV/TV is indicated below. Over, BMSCs transduced with Lenti-DLX2 OE; control, BMSCs transduced with Lenti-CTRL. **d**–**g** Analysis of the bone volume/tissue volume (BV/TV), bone mineral density (BMD), trabecular number (Tb.N), and trabecular spacing (Tb.Sp) in the respective groups. Statistical significance was determined as described in Fig. 1; *n* = 6, *P < 0.05, **P < 0.01

To investigate whether *Dlx2* overexpression could affect osteogenesis in vivo, we subcutaneously implanted the BMSCs/β-tricalcium phosphate (β-TCP) constructs into nude mice. The whole implanted tissues were then analyzed with microscopic computed tomography (micro-CT) examination 6- or 8-weeks after implantation (Fig. 3a). Micro-CT revealed new bone formation in both control and *Dlx2* overexpression groups (Fig. 3b, c). However, quantitative morphometric analysis showed that bone volume/tissue volume (BV/TV) in the *Dlx2*-overexpressing group (17.81% ± 1.23% and 23.64% ± 1.71% at week 6 and week 8, respectively) was significantly higher than that in the control group (12.91% ± 1.16% and 17.04% ± 1.62% at week 6 and week 8, respectively) (*P* < 0.05) (Fig. 3d). Similarly, the *Dlx2*-overexpressing group showed higher bone mineral density (BMD) of the newly formed bone ((1.477 ± 0.097) g· cm^−3^ and (1.550 ± 0.121) g· cm^−3^ at week 6 and week 8, respectively) than the control group ((1.055 ± 0.072) g· cm^−3^ and (1.107 ± 0.098) g· cm^−3^ at week 6 and week 8, respectively) (Fig. 3e). Besides, trabecular number (Tb.N) in the *Dlx2*-overexpressing group ((1.362 ± 0.110) g· cm^−3^ and (1.713 ± 0.129) g· cm^−3^ at week 6 and 8, respectively) was significantly higher than that in the control group ((0.979 ± 0.086) g· cm^−3^ and (1.232 ± 0.097) g· cm^−3^ at week 6 and 8, respectively) (Fig. 3f). Additionally, the *Dlx2*-overexpressing group exhibited decreased trabecular space (Tb.Sp) ((0.634 ± 0.059) mm and (0.482 ± 0.033) mm at week 6 and 8, respectively) when compared to the control group ((0.885 ± 0.079) mm and (0.672 ± 0.044) mm at week 6 and 8, respectively) (Fig. 3g). Taken together, these results demonstrate that overexpression of *Dlx2* in BMSCs substantially triggers osteogenic differentiation and improves bone formation in vivo, defining a pivotal role of Dlx2 in osteogenic differentiation.

### Effect of forced overexpression of Dlx2 on the expression of osteogenic genes in BMSCs

**Fig. 4:**
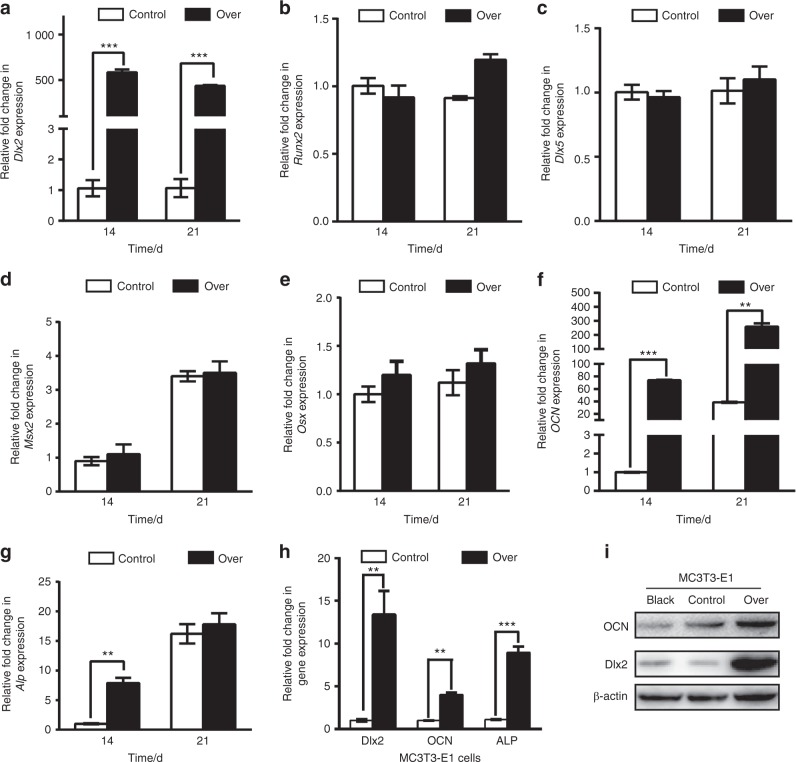
*Dlx2* overexpression in BMSCs has no impact on *Runx2*, *Dlx5*, *Msx2 and* *Osx*expression. RT-qPCR analysis was performed to evaluate the expression levels of *Dlx2* (**a**), *Runx2* (**b**), *Dlx5* (**c**), *Msx2* (**d**), *Osx* (**e**), *OCN* (F), and *Alp* (**g**) in BMSCs transduced with Lenti-DLX2 OE (over) or Lenti-CTRL (control) at 14 and 21 days after osteogenic induction. *Gapdh* was used as an internal control. Statistical significance was determined as described in Fig. 1. **h** RT-qPCR analysis was used to evaluate the expression level of *OCN* and *Alp* upon forced overexpression of *Dlx2* in MC3T3-E1 cells. **i** Western blot analysis was performed to measure the protein levels of OCN upon forced overexpression of *Dlx2* in MC3T3-E1. β-Actin was used as an internal control. Over, BMSCs transduced with Lenti-DLX2 OE; control, BMSCs transduced with Lenti-CTRL

To gain an insight into the mechanism of *Dlx2*-induced osteogenesis, we examined the expression of a panel of osteogenesis-associated genes by RT-qPCR in *Dlx2*-overexpressing BMSCs and control BMSCs cultured in OIM. As shown in Fig. 4a, RT-qPCR analysis revealed that transduction of Lenti-DLX2 OE into BMSCs resulted in a 526.2-fold and 481.3-fold increase in *Dlx2* expression at days 14 and 21 of osteogenic differentiation, respectively. Unexpectedly, there was no difference in *Runx2*, *Dlx5*, *Msx2*, and *Osx* expression between *Dlx2*-overexpressed BMSCs and mock-transduced BMSCs at 14 and 21 days after osteogenic induction (Fig. 4b–e) nor at 1, 2, and 7 days after osteogenic induction (data not shown), indicating that these genes might not be involved in the *Dlx2*-induced promotion of osteogenesis. Importantly, forced overexpression of *Dlx2* led to upregulation of *OCN* at days 14 and 21 after osteogenic induction and upregulation of *Alp* at day 14 (Fig. 4f, g), consistent with the ALP staining and Alizarin red staining results (Fig. 2a–c). In summary, we found, for the first time, that forced overexpression of *Dlx2* in BMSCs leads to upregulation of *Alp* and *OCN*, instead of *Runx2*, *Dlx5*, *Msx2*, and *Osx*.

### Dlx2 overexpression leads to increased expression of *OCN* and *Alp* in MC3T3-E1 cells

We next tested whether *Dlx2* overexpression could also lead to upregulation of *OCN* and *Alp* in other osteoblast precursor cell lines. Compared with mock-transduced cells, we observed enhanced mRNA and protein levels of *OCN* in *Dlx2*-overexpressing MC3T3-E1 cells after osteogenic induction (Fig. 4h, i). *Dlx2*-overexpressing MC3T3-E1 cells also exhibited higher *Alp* transcription (Fig. 4h) and enhanced ALP activity (Fig. S1A). Moreover, as in BMSCs, we observed no significant difference in *Msx2*, *Dlx5*, *Runx2*, and *Osx* expression after Lenti-DLX2 OE transduction in MC3T3-E1 cells after osteogenic induction, as shown in the Figure S2.

Taken together, these data demonstrate that forced overexpression of *Dlx2* induces upregulation of *OCN* and *Alp* upon osteogenic induction both in BMSCs and MC3T3-E1 cell lines. Given that the expression levels of *Runx2*, *Dlx5*, *Msx2*, and *Osx* remained unchanged upon *Dlx2* overexpression, we speculate that *OCN* and *Alp* are the direct target genes of Dlx2.

### Characterization of the mouse OCN promoter and identification of its Dlx2-response element

Previous genetic studies have shown that through direct binding to the promoter of *OCN*, homeodomain (HD) proteins Msx2, Dlx3, and Dlx5 regulate the expression of *OCN* in osteogenic cells. Dlx3 binds the *OCN* promoter to stimulate its expression, while the binding of Msx2 and the recruitment of Dlx5 represses *OCN* expression. Moreover, Dlx5 and Msx2 regulate *Alp* expression by directly binding to its promoter.^15,16^ Considering that Dlx2, an HD protein, shares strong sequence similarity with Dlx5 and Dlx3, we speculated that Dlx2 might also be involved in the regulation of *OCN* and *Alp* transcription in osteogenic cells by binding to their promoters.

**Fig. 5:**
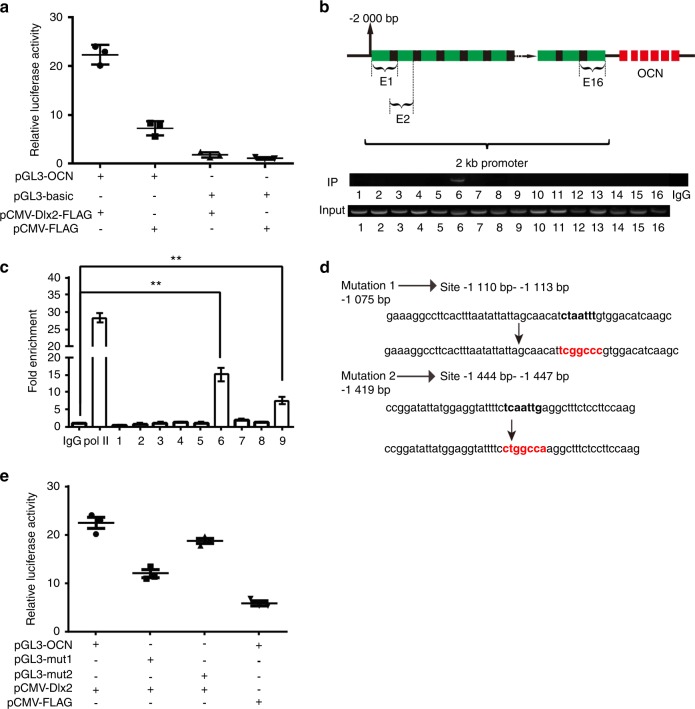
Characterization of the mouse *OCN* promoter and identification of its Dlx2-response elements. **a** The basal luciferase activity of the whole *OCN* promoter construct (pGL3-OCN) and that of the empty (pGL3-basic) construct in MC3T3-E1 cells was determined by transfecting the cells with each promoter reporter construct along with the *Dlx2* overexpression vector (pCMV-Dlx2-FLAG) or the empty vector (pCMV-FLAG). Cells were then harvested 24 h after the transfection, and luciferase activity was measured and normalized to the protein concentration in the cell lysate. **b** ChIP analysis was performed to determine the Dlx2-response elements in the *OCN* promoter. MC3T3-E1 cells were transfected with pCMV-Dlx2-FLAG. Semi-quantitative PCR was performed using overlapping and closely spaced primer pairs to dissect the whole *OCN* promoter region into 16 short (~ 175 bp) overlapping parts for identification of the bound protein. Normal IgG (2 μg) was used as control. The PCR products were then separated by electrophoresis through a 2% agarose gel. **c** ChIP analysis followed by RT-qPCR was performed using the same primers described in **b**. Statistical significance was determined as described in Fig. 1. **d** The sequences of the nucleotides whose sequences contain the AATT element in the *OCN* promoter and the sequences of two mutants. The mutant binding sites are marked in red, and the putative Dlx2-binding sites are indicated in parentheses. **e** The luciferase activity of wild-type *OCN* promoter constructs (pGL3-OCN) and the mutated ones (pGL3-mut1 and pGL3-mut2) were determined by transfecting these vectors into MC3T3-E1 cells along with pCMV-Dlx2-FLAG or pCMV-FLAG. The *OCN* promoter construct bearing approximately −2000 to 0 bp region was subjected to site-directed mutagenesis to substitute the AATT sequence (pGL3-OCN) with either mutation1 (pGL3-mut1) or mutation2 (pGL3-mut2). The luciferase activity was measured 24 h later

We first determined whether *OCN* is directly regulated by Dlx2, we analyzed its promoter in MC3T3-E1 cells. We inserted the whole promoter region of *OCN* into the pGL3-basic plasmid, generating pGL3-OCN, and transferred this vector into MC3T3-E1 cells along with pCMV-Dlx2-FLAG to allow the overexpression of *Dlx2*. As shown in Fig. 5a, we observed an ~3.2-fold increase in the transcriptional activity of pGL3-OCN after introduction of pCMV-Dlx2-FLAG into MC3T3-E1 cells, suggesting that the *OCN* promoter contains at least one Dlx2-response element (RE).

To further identify the Dlx2-RE(s) in the *OCN* promoter, we performed ChIP analysis in MC3T3-E1 cells transfected with pCMV-Dlx2-FLAG. Semi-quantitative PCR analysis showed one strong signal in the E6 region (−1 311 bp to −1 175 bp) matching the predicted ~175 bp size of the E6 PCR product (Fig. 5b). Considering the low sensitivity of semi-quantitative PCR, we next carried out RT-qPCR. Consistent with the above findings, qPCR also revealed that Dlx2 was highly enriched at region E6 (−1 311 to −1 175 bp) and at region E9 (−1 073 bp to – 932 bp) (Fig. 5c). The qPCR results of region E10 to region E16 are not shown since the signals at these regions were as weak as the negative control in semi-quantitative PCR results. In addition, the occupancy of Dlx2 in the *OCN* promoter was correlated with increased transcription represented by elevated occupancy of RNA polymerase II (Pol II) (Fig. 5c), while the negative control (cells introduced with pCMV-FLAG) showed very weak signal (data not shown). These results indicate that there is a Dlx2-RE in the *OCN* promoter in region E6 (in primer set 6) and E9 (in primer set 9). Consistent with this, bioinformatics analysis (JASPAR database) indicated that the promoter region of *OCN* contains potential Dlx2-REs (−1 447 bp to −1 444 bp and −1 113 bp to −1 110 bp), both of which have the ATTA sequence.

We next checked whether Dlx2 can directly bind to the two predicted Dlx2-REs in the *OCN* promoter. To do this, we inserted mutated *OCN* promoter into the pGL3-basic plasmid in which the ATTA sequence was partly mutated, generating pGL3-mut1 (upstream RE mutant) and pGL3-mut2 (downstream RE mutant) (Fig. 5d). As shown in Fig. 5e, we found that mutation of either the upstream RE or downstream RE abrogated the ability of Dlx2 to regulate its activity. These observations demonstrate that in MC3T3-E1 cells, Dlx2 directly binds to the upstream RE and downstream RE in the *OCN* promoter to positively regulate its transcription.

### Dlx2 upregulates *Alp* expression by directly binding to its promoter

**Fig. 6:**
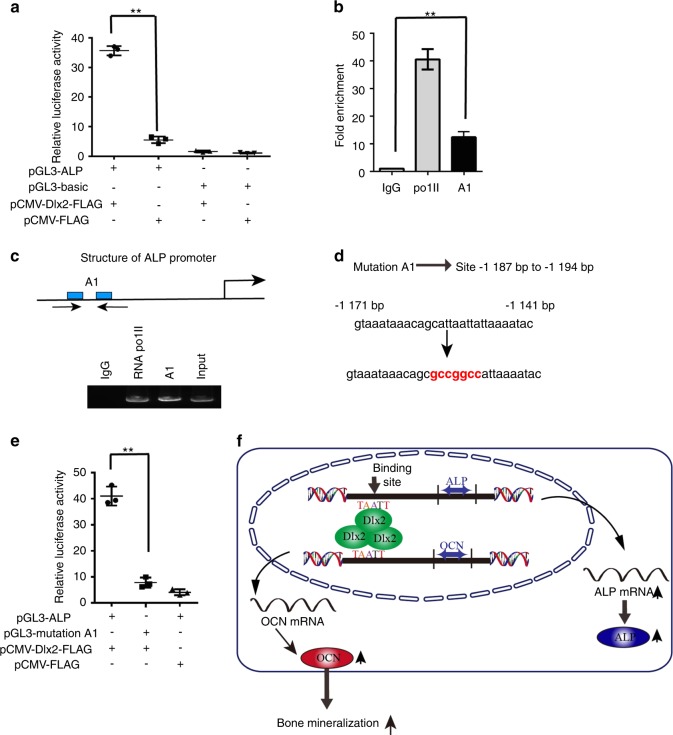
Characterization of the mouse *Alp* promoter and identification of its Dlx2-response element. **a** The basal luciferase activity of the whole *Alp* promoter construct (pGL3-ALP) and that of the empty (pGL3-basic) construct in MC3T3-E1 cells was determined by transfecting the cells with each promoter reporter construct along with the *Dlx2* overexpression vector (pCMV-Dlx2-FLAG) or the empty vector (pCMV-FLAG). Cells were then harvested 24 h after the transfection and luciferase activity was measured and normalized to the protein concentration in the cell lysate. **b** ChIP-qPCR analysis was performed to determine the Dlx2-response elements in the *Alp* promoter. MC3T3-E1 cells were transfected with pCMV-Dlx2-FLAG. Normal IgG (2 μg) was used as control. Pol II was used as a positive control. **c** ChIP analysis followed by RT-PCR was performed using the same primers described in **b**. **d** The sequence of the nucleotides whose sequences contain attaatt sequence in the *Alp* promoter and the sequence of the mutant. The mutant binding sites are marked in red and the putative Dlx2-binding sites are indicated in parentheses. **e** The luciferase activity of wild-type *Alp* promoter constructs (pGL3-ALP) and the mutated ones (pGL3-mutationA1) were determined by transfecting these vectors into MC3T3-E1 cells along with pCMV-Dlx2-FLAG or pCMV-FLAG. The *Alp* promoter construct bearing ~−2000 to 0 bp region was subjected to site-directed mutagenesis to substitute the original sequence (pGL3-ALP) with mutation (pGL3-mutationA1). The luciferase activity was measured 24 h later. **f** Schematic illustration of the regulation of *OCN* and *Alp* by Dlx2

Next, we tried to investigate whether *Alp* was regulated by Dlx2 in a similar way by binding to its promoter. To verify this transcriptional regulation, we first cloned and inserted the whole promoter region of *Alp* into the pGL3-basic plasmid, generating pGL3-ALP. This vector was introduced into MC3T3-E1 cells along with pCMV-Dlx2-FLAG to allow overexpression of Dlx2. As shown in Fig. 6a, we observed an ~6.7-fold increase in the transcriptional activity of pGL3-ALP after introduction of pCMV-Dlx2-FLAG into MC3T3-E1 cells, indicating that there is at least one Dlx2-RE in the *Alp* promoter. Supporting this notion is that bioinformatics analysis (JASPAR database) indicated that the promoter region of *Alp* contains potential Dlx2-RE (−1 194 bp to −1 187 bp, region A1).

Next, we checked whether Dlx2 can bind directly to the predicted Dlx2-REs (A1) in the *Alp* promoter. ChIP analysis was performed in MC3T3-E1 cells transfected with pCMV-Dlx2-FLAG or pCMV-FLAG, and primer set was designed to amplify the region A1. As shown in Fig. 6b, c, both qPCR and semi-quantitative PCR revealed that Dlx2 was highly enriched in region A1. In addition, elevated occupancy of Pol II in the *Alp* promoter was associated with increased occupancy of Dlx2 in this region, while the negative control (MC3T3-E1 cells introduced with pCMV-FLAG) showed very weak signal (data not shown).

To determine whether A1 is the Dlx2-binding site in the *Alp* promoter, we carried out site-directed mutagenesis in A1 region. We inserted mutated *Alp* promoter into the pGL3-basic plasmid, generating pGL3-mutationA1 (Fig. 6d). As shown in Fig. 6e, we found that mutation of A1 significantly attenuated the ability of Dlx2 to regulate its activity. These findings demonstrated that Dlx2 directly binds to region A1 in the *Alp* promoter to positively regulate its expression (Fig. 6e). Taken together, all these data proved that Dlx2 promotes *OCN* and *Alp* expression by directly binding to their promoters, and then regulates osteogenic differentiation in vitro and accelerates bone formation in vivo.

## Discussion

Dlx gene family plays a critical role in osteogenesis. Previous study showed that *Dlx2*, *Dlx5*, and *Dlx6* are upregulated in immature osteoblasts, while the expression of *Dlx3* is elevated in differentiated osteoblasts and osteocytes. Dlx3 and Dlx5 potently transactivates osteoblastic marker genes^15^; Dlx6 also has stimulatory effects on osteogenic differentiation.^17^ These findings indicate that as a member of Dlx gene family, *Dlx2* may be also involved in the osteogenic differentiation.

**Table 1:** Primers used in this study

Primer	Sequence	Details	Reference
Primers for RT-qPCR			
ep Dlx2 FW	CATGGGCTCCTACCAGTACCAC	qPCR primer for *Dlx2*	^8^
ep Dlx2 RV	TCGGATTTCAGGCTCAAGGTC	qPCR primer for *Dlx2*	^8^
ep Gapdh FW	GGTGAAGGTCGGTGTGAACG	qPCR primer for *Gapdh*	^8^
ep Gapdh RV	CTCGCTCCTGGAAGATGGTG	qPCR primer for *Gapdh*	^8^
ep ALP FW	TGGGCATTGTGACTACCACTCGG	qPCR primer for *Alp*	^8^
ep ALP RV	CCTCTGGTGGCATCTCGTTATCC	qPCR primer for *Alp*	^8^
ep OCN FW	GGACCATCTTTCTGCTCACTCTG	qPCR primer for *Ocn*	^8^
ep OCN RV	GTTCACTACCTTATTGCCCTCCTG	qPCR primer for *Ocn*	^8^
ep Runx2 FW	AACTTCCTGTGCTCCGTGCTG	qPCR primer for *Runx2*	^8^
ep Runx2 Rv	TCGTTGAACCTGGCTACTTGG	qPCR primer for *Runx2*	^8^
ep Msx2 FW	GGAGCACCGTGGATACAGGA	qPCR primer for *Msx2*	^8^
ep Msx2 RV	AGGCTAGAAGCTGGGATGTGG	qPCR primer for *Msx2*	^8^
ep OSX FW	CCCTTCTCAAGCACCAATGG	qPCR primer for *Osx*	^41^
ep OSX RV	AAGGGTGGGTAGTCATTTGCATA	qPCR primer for *Osx*	^41^
Primers for ChIP analysis			
Primer-F16	gaggctgagagagagagcaca	For ChIP-qPCR	This study
Primer-R16	tgctgtggtaggtgattgc	For ChIP-qPCR	This study
Primer-F15	gcagacactgaaaatcacagg	For ChIP-qPCR	This study
Primer-R15	tgtgctctctctctcagcctc	For ChIP-qPCR	This study
Primer-F14	cataaaactaaccaggacactcc	For ChIP-qPCR	This study
Primer-R14	gcctgtgattttcagtgtctgc	For ChIP-qPCR	This study
Primer-F13	ccacaatgggctaggctct	For ChIP-qPCR	This study
Primer-R13	catccagtgggggtgtgt	For ChIP-qPCR	This study
Primer-F12	acacaagcagggctagaacc	For ChIP-qPCR	This study
Primer-R12	agcccattgtgggagctac	For ChIP-qPCR	This study
Primer-F11	ttgtggacatcaagcggg	For ChIP-qPCR	This study
Primer-R11	aggttctagccctgcttgtg	For ChIP-qPCR	This study
Primer-F10	tttcatttccacctagagcaag	For ChIP-qPCR	This study
Primer-R10	tcgggggtatctggtttcag	For ChIP-qPCR	This study
Primer-F9	gtttggcatggagcctttg	For ChIP-qPCR	This study
Primer-R9	tgtgttacagtcacttgctctagg	For ChIP-qPCR	This study
Primer-F8	gccctcttctagtgtgtctgaa	For ChIP-qPCR	This study
Primer-R8	cccaagttcaaaggctccat	For ChIP-qPCR	This study
Primer-F7	cacagttggactggggaggt	For ChIP-qPCR	This study
Primer-R7	cttcagacacactagaagagggc	For ChIP-qPCR	This study
Primer-F6	tcaccagcgcaaatcacac	For ChIP-qPCR	This study
Primer-R6	ctgtgtgctttcttattcacctacc	For ChIP-qPCR	This study
Primer-F5	aagggaaacaccacccactc	For ChIP-qPCR	This study
Primer-R5	atttgcgctggtgaggac	For ChIP-qPCR	This study
Primer-F4	cctccactgcctcaagaact	For ChIP-qPCR	This study
Primer-R4	aaggctggaaaggagtgggt	For ChIP-qPCR	This study
Primer-F3	acagaaggcaggtcagctaca	For ChIP-qPCR	This study
Primer-R3	gggaagagtaaggtggaggtt	For ChIP-qPCR	This study
Primer-F2	ttccccctagccgaacaag	For ChIP-qPCR	This study
Primer-R2	acctgccttctgtgatcctct	For ChIP-qPCR	This study
Primer-F1	agaggatcacagaaggcaggt	For ChIP-qPCR	This study
Primer-R1	aaaggatgctgtggttggtgattg	For ChIP-qPCR	This study
Primer-A1F	tgcctgggtttgttttcatttgt	For ChIP-qPCR	This study
Primer-A1R	caagggaaatttcctagcac	For ChIP-qPCR	This study

Dlx2, a member of vertebrate Dlx gene family, is expressed in the epithelium and mesenchyme of the mandible and maxilla.^18^ Previous studies have shown that newborn *Dlx2*^−/−^ mice die immediately after birth and have abnormal craniofacial bones originating from the first branchial arch maxillary process,^9^ while *Dlx2* overexpression in cranial neural crest cell (CNCC) leads to premaxillary hypoplasia and spinal deformities in mice.^9^ Although *Dlx2* knockout and overexpression models demonstrate a crucial role of Dlx2 in promoting skeleton formation, the molecular and cellular mechanisms underlying the regulation of osteogenic differentiation by Dlx2 still remain unclear.

Here, we present evidence that forced overexpression of *Dlx2* enhances the osteogenic differentiation potential of mouse BMSCs and MC3T3-E1 in vitro and accelerates bone formation in vivo by directly regulating *OCN* and *Alp*. Our finding is consistent with the previous study in SCAPs that overexpression of Dlx2 also enhanced osteogenic differentiation.^19^ SCAPs are mesenchymal-like stem cells that are able to differentiate into multiple linages, including odontoblastic and osteoblastic lineage, and do not undergo adipogenic differentiation, while BMSCs are able to undergo osteogenic, chondrogenic, and adipogenic differentiation.^20^ Relative to BMSCs, SCAPs display elevated secretion of proteins involved in metabolic processes, chemokines, and neutrophins, whereas BMSCs secret much more proangiogenic factors and ECM proteins. Therefore, overexpression of *Dlx2* in SCAPs and BMSCs could be used in dentin regeneration and bone formation, respectively.^21^

The rodent *Dlx5* and *Dlx2* HD transcription factors are critical for bone development. During osteogenesis, Dlx5 upregulates *Alp* expression while suppresses *OCN* transcription.^22^ On the contrary, we found that Dlx2 transactivates *OCN* and *ALP* by directly binding to their promoters. Moreover, we identified two Dlx2-REs in *OCN* promoter, and one Dlx2-RE in *Alp* promoter involved in Dlx2-induced *Alp* expression. These findings demonstrate that Dlx2 is a crucial regulator regulating the osteogenic differentiation potential of both mesenchymal stem cells and osteogenic cells.

BMP-2 is one of the most important cytokines promoting differentiation of mesenchymal cells into osteoblasts.^23^ Stimulated by BMP-2, the transcription factors Dlx5, Msx2, and Runx2 work coordinately to regulate osteogenic differentiation.^24^ Both Runx2 and Dlx3 positively regulate *OCN* expression while Dlx5 represses its expression; Dlx5 stimulates ALP expression, whereas Msx2 depresses its expression.^25^ At the onset of osteogenic differentiation, Msx2 is released from the promoter of OCN, while Dlx3, Dlx5, and Runx2 are recruited. The released Msx2 then binds to the *Alp* promoter to upregulate its expression. At a later stage of osteogenic differentiation, during matrix mineralization, Dlx5 replaces Dlx3 to regulate OCN expression.^26^ Runx2 and Osx are another key transcription factors that are necessary for osteogenesis.^27^ After differentiating into pre-osteoblasts, Runx2 and Osx promote the cells to produce bone matrix. Dlx gene family is involved in the regulation of *Runx2* and OSX transcription. Dlx5 induces expression of Runx2 and Osx, which work sequentially to stimulate the expression of *OCN* and *Alp*. Dlx3 also contributes to the activation of Runx2 during osteogenic differentiation.^28^ These findings indicate that Dlx gene family plays a crucial role in expression of osteogenic-associated genes. The results of the present study showed that overexpression of Dlx2 showed no significant effects on *Runx2*, *Msx2*, and *Dlx5* expression upon osteogenic induction, but stimulated *OCN* and *Alp* expression, indicating that Runx2, Msx2, and Dlx5 may not participate in Dlx2-induced osteogenesis; Dlx2 may directly upregulate *OCN* and *Alp* to promote osteogenic differentiation.

*Alp* and *OCN* are two key marker genes of osteoblastic cells. ALP plays a critical role in early osteogenesis and hydrolyzes various types of phosphates to promote cell maturation and calcification, while OCN promotes the later stage of osteogenesis through combining with minerals.^29,30^ Both Dlx3 and Dlx5 directly upregulate *Alp* expression, while OCN is activated by Dlx3 but suppressed by Dlx5.^16,31^ Moreover, forced overexpression of *Dlx5* in BMSCs led to a reduction in the mineralized matrix deposition, and impaired the ability of these cells to develop to the final stages of osteogenesis, and severely affected in vivo bone formation in immunodeficient mice.^13^ Although Dlx2 shares a strong sequence similarity with Dlx5, we found that Dlx2 positively regulates both *Alp* and *OCN* expression in BMSCs and MC3T3-E1 cells.

A previous study showed that loss of Dlx1/2^−/−^ leads to abnormal bone formation of the upper jaw, while Dlx5/6^−/−^ deficient mice exhibit profound abnormalities of the lower jaw tissue.^22^ However, piles of studies revealed that Dlx5 is the master regulator of osteogenic differentiation, since it directly controls the transcription of multiple osteogenic-associated genes, including *Alp*, *OCN*, *Runx2*, *OSX*, and *Smads* family, affecting the whole process of bone formation.^32,33^ Therefore, Dlx5 is also involved in the development of the upper jaw, but may be not as important as Dlx2. On the other hand, the maxilla is only formed by intramembranous ossification of the craniofacial mesenchyme, while the mandible can be formed by both intramembranous and endochondral ossification.^34^ Our previous study has shown that Dlx2 is involved in endochondral ossification.^9,35^ Therefore, Dlx2 is also involved in the bone formation of lower jaw, but is not as important as Dlx5. Together, both Dlx2 and Dlx5 are involved in the development of mandible and maxilla. Considering the fact that a variety of homeobox genes work coordinately during the bone formation,^36^ further investigations are required to find out how Dlx2 is involved during the bone formation in mandible and maxilla.

In conclusion, our data demonstrated for the first time that forced overexpression of *Dlx2* enhances the osteogenic differentiation potential of BMSCs and MC3T3-E1 cells by directly upregulating *OCN* and *Alp* (Fig. 6f). We also presented evidence that there are Dlx2-REs in mouse *OCN* and *Alp* promoter that mediate the regulation of Dlx2 on *OCN* or *Alp* expression. This study may present a promising future strategy for the treatment of bone defects with *Dlx2*-overexpressing BMSCs.

## Material and methods

### Isolation and culture of mouse BMSCs

All animal experiments were performed according to guidelines of the Institutional Animal Care and Use Committees of the Shanghai Ninth People’s Hospital, Shanghai Jiao Tong University School of Medicine. All experimental protocols were reviewed and approved by the Institutional Animal Care and Use Committees of the Shanghai Ninth People’s Hospital, Shanghai Jiao Tong University School of Medicine, Shanghai, China. BMSCs were isolated from the tibias of 6-week-old male C57/BL6 mice ((10 ± 0.5) g) and cultured according to previous study.^37^

An MC3T3-E1 cell line was obtained from the Cell Bank of the Chinese Academy of Science (Shanghai, China), C3H10 T1/2 cell line from American Type Culture Collection (Rockville, MD, USA), and a Human embryonic kidney 293T (HEK 293T) cell line from American Type Culture Collection (Rockville, MD, USA). The MC3T3-E1, C3H10 T1/2 cell line, and HEK 293T cells were cultured as described previously.^38,39^ OIM contained 50 mg· L^−1^ ascorbic acid, 1 × 10^−7^ mol· L^−1^ dexamethasone, and 50 mg· L^−1^ β-glycerophosphate plus α-MEM (Sigma-Aldrich Corp. (St. Louis, MO, USA)). HEK 293T cell line was utilized for packaging viral constructs.

### Lentiviral construction and transduction

The lentiviral expression system overexpressing *Dlx2* was termed as Lenti-Dlx2 OE. The open reading frame of mice *Dlx2* (NM_010054) was synthesized and cloned into pL/IRES/GFP plasmid (Novobio, Shanghai, China) for generating pL/IRES/GFP-DLX2. The empty lentiviral expression system without insertion was termed as Lenti-CTRL and used as the control. 293T cells were then transfected with plasmids listed above. The transfection and lentiviral transduction was done as described previously.^40,41^

### ALP, Alizarin red staining, and semi-quantitative analysis

Transduced BMSCs or MC3T3-E1 cells were first cultured in OIM for 14 or 21 days. ALP and Alizarin red staining were carried out as described previously.^40^ ALP staining was carried out with BCIP/NBT Alkaline Phosphatase Color Development Kit (Beyotime Institute of Biotechnology, China), and semi-quantitative analysis of ALP activity was performed using *p*-nitrophenyl phosphate (*p*-NPP) (Sigma-Aldrich) as the substrate. For Alizarin red staining, cells were first fixed with 70% ethanol. Afterward, the fixed cells were stained with 2% Alizarin Red (Sigma-Aldrich), according to the previous study.^19^

### Semi-quantitative RT-PCR, RT-qPCR, and Western blot analysis

RT-PCR, RT-qPCR, and western blot analysis were performed as described previously.^40^ Total RNA was extracted from cultured cells using TRIzol RNA Isolation reagent (Takara, Tokyo, Japan), according to the manufacturer’s instruction. Three independent cultures were used for RNA preparations. First-strand cDNA was generated with High-Capacity cDNA Reverse Transcription Kit, (Applied Biosystems, San Diego, CA), and one microliter of each RT reaction mixture was amplified with Ex Taq DNA polymerase (Takara, Tokyo, Japan). As for RT-qPCR, cDNA was amplified using premix SYBR Green Ex Taq reagent kit (DRR820A, Takara) with a STEP ONE PLUS real-time PCR system (Applied Biosystems, Forster City, CA), according to the manufacturer’s instruction. All the primers used in this study are listed in Table 1. As for western blotting, anti-Dlx2 (1:800; ab85995, Abcam, Cambridge, UK), anti-OCN (1:1 000; ab93876, Abcam), and anti-β-actin (1:3 000; EPR16769, Abcam, Cambridge, UK) were used for the detection of Dlx2, OCN, and β-actin, respectively. The secondary antibodies used this study were bought from Sigma-Aldrich and conjugated to horseradish peroxidase (anti-rabbit (1:5 000, A0545) or anti-mouse (1:5 000, SAB3701214)).

### In vivo osteogenic differentiation

The osteogenic differentiation potential of transduced BMSCs was evaluated with in vivo ectopic bone formation analysis, as described previously.^13^ Briefly, osteogenic-induced cells of passage 3 were injected into the β-TCP (Shanghai Rebone Biomaterials Co., Ltd., Shanghai, China) with a syringe, and appropriate volume of OIM was added to cover the BMSCs/ β-TCP constructs. After 7 days of culturing in vitro, the constructs were implanted subcutaneously in the back of immunocompromised female nude mice (CD-1 Nu/Nu, 10-week-old, Charles River). At each time point (6 and 8 weeks after implantation), six mice for each group were sacrificed with an overdose of pentobarbital (210 mg/kg intraperitoneally) to retrieve the BMSCs/β-TCP constructs. Micro-CT images of each cellular construct were taken with a micro-CT system (SMX-100CT-SV3; Shimadzu, Japan). Radiological density of each cellular construct was measured in Hounsfield density units.

### Luciferase assay

The whole mouse *OCN* promoter (2000 bp) was subcloned into the pGL3 basic vector (Promega), generating pGL3-OCN. To produce constructs that contain mutation in E6 region, we carried out mutagenic PCR with Mt1 primer and Mt2 primer, generating pGL3-mut1. Likewise, Mt3 and Mt4 primers were used for generating pGL3-mut2 (Table 1). The open reading frame of mice *Dlx2* (NM_010054) was synthesized and cloned into pCMV-FLAG, generating pCMV-Dlx2-FLAG for *Dlx2* overexpressing. Transfection assay was performed as described previously.^31^ As for each transfection assay, 0.5 μg of the *Dlx2* overexpression vector (pCMV-Dlx2-FLAG) or pCMV-FLAG and 0.5 μg of the luciferase reporter vector were transfected into MC3T3-E1 cells. Similarly, we inserted the whole *Alp* promoter into the pGL3-basic vectors, generating pGL3-ALP, and produced a vector that contains mutated *Alp* promoter, generating pGL3-mutationA1. Afterward, pCMV-Dlx2-FLAG or pCMV-FLAG was introduced into MC3T3-E1 cells along with pGL3-ALP or pGL3-mutationA1.

### ChIP analysis

ChIP analysis was carried out according to the standard protocol.^15^ MC3T3-E1 cells transfected with pCMV-Dlx2-FLAG or pCMV-FLAG were fixed with 1% formaldehyde and this cross-linking was quenched with glycine (0.125 mol· L^−1^ for final concentration). After the cells were lysed and homogenized with a Dounce homogenizer, the nuclei were collected by centrifuging. The nuclei pellet was then resuspended in sonication buffer followed by sonication. Chromatin were incubated with anti-FLAG antibodies (F3165, Sigma-Aldrich), mouse IgG and anti-RNA-polymerase II (PLA0292, Sigma-Aldrich), and this immune complex were incubated with protein G sepharose (17-0618-01, GE Amersham). The DNA fragments in this immune complex were purified with phenol–chloroform extraction. ChIP samples were then analyzed with RT-PCR and RT-qPCR. Pol II was used as a positive control, as described in a previous study.^15^ The primers used in ChIP analysis are listed in Table 1.

## Supplementary information

supplemental Figure legends
