# Supplementary material for: Overexpression of Dlx2 enhances osteogenic differentiation of BMSCs and MC3T3-E1 cells via direct upregulation of Osteocalcin and Alp
Source: Int J Oral Sci. 2019 Mar 18;11(2):12. doi: 10.1038/s41368-019-0046-1 (PMC6421343; doi:10.1038/s41368-019-0046-1)
Supplement: Supplementary file 1 — supplemental Figure legends [file 41368_2019_46_MOESM1_ESM.docx]

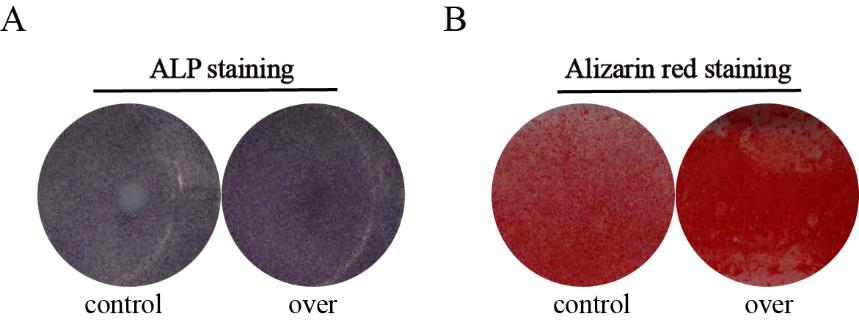


Fig. S1 Forced overexpression of *Dlx2* enhanced osteogenesis of MC3T3-E1 in vitro. (A) ALP staining of MC3T3-E1 cells on days 14 upon osteogenic induction. (B) Alizarin red staining of MC3T3-E1 cells on days 21 upon osteogenic induction. Over: *Dlx2*-overexpressing cells; Control: cells introduced with mock vector.


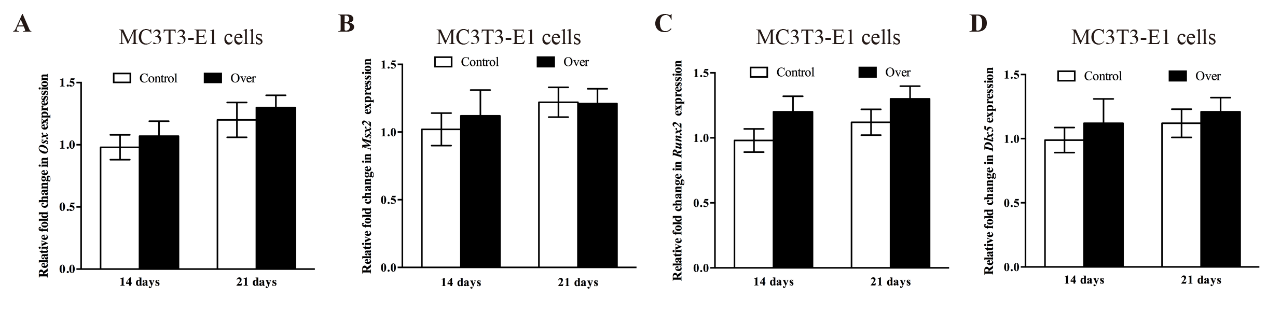


Fig. S2 *Dlx2* overexpression had no impact on *Osx*, *Msx2*, *Runx2* and *Dlx5* expression in osteogenic cells. RT-qPCR analysis was performed to evaluated expression levels of *Osx* (A, E), *Msx2* (B, F), *Runx2* (C, G) and *Dlx5* (D, H) in MC3T3-E1 cells. *Gapdh* was used as an internal control. Statistical significance was determined as described in Figure 1.
